# Knowledge, attitudes, and practices of cervical cancer prevention and pap smears in two low-income communities in Lima, Peru

**DOI:** 10.1186/s12905-021-01291-8

**Published:** 2021-04-21

**Authors:** Thomas T. Miles, Amy R. Riley-Powell, Gwenyth O. Lee, Esther E. Gotlieb, Gabriela C. Barth, Emma Q. Tran, Katherine Ortiz, Cynthia Anticona Huaynate, Lilia Cabrera, Patti E. Gravitt, Richard A. Oberhelman, Valerie A. Paz-Soldan

**Affiliations:** 1 Global Community Health and Behavioral Sciences Department, Tulane University School of Public Health and Tropical Medicine, 1440 Canal St., Suite 2200, New Orleans, LA 70112 USA; 2 Department of Epidemiology, University of Michigan School of Public Health, 1415 Washington Heights, Ann Arbor, MI 48109-2029 USA; 3 Tulane University, 6823 St Charles Ave, New Orleans, LA 70118 USA; 4 Asociación Benéfica PRISMA, Lima, Peru - Carlos Gonzales 251, San Miguel, 15088 USA; 5 Department of Epidemiology and Public Health, School of Medicine, University of Maryland, Baltimore, 655 W. Baltimore Street, Baltimore, MD 21201 USA

**Keywords:** Cervical cancer, KAP, Peru, Shantytown, Peri-urban

## Abstract

**Background:**

Cervical cancer is a leading cause of death among Peruvian women. Barriers at multiple levels impact effective screening and treatment, including a lack of knowledge about cervical cancer and how regular screening can reduce morbidity and mortality through earlier detection. The aim of this study is to assess knowledge, attitudes, and practices regarding cervical cancer and its prevention in the peri-urban communities of Oasis and Pampas in southern Lima, Peru that can be used to inform future campaigns about cervical cancer prevention.

**Methods:**

A cross-sectional survey that included several open-ended questions was administered to women in Pampas and Oasis between 2015 and 2016 to evaluate the knowledge, attitudes, and practices regarding cervical cancer and Pap smears.

**Results:**

In total, 224 women were interviewed. Knowledge about cervical cancer and Pap smears was high, and attitudes were predominantly positive among most participants. Most participants knew how often they should get Pap smears (89.7%), when to begin seeking screening (74.6%), knew the price of a Pap smear (61.9%), and felt Pap smears were important for their health (70.1%). About one third (29.5%) of premenopausal women reported receiving a Pap smear in the last year. However, open ended questions revealed some knowledge gaps around Pap smears, as well as some stigma associated to Human Papilloma Virus (HPV) infection.

**Conclusion:**

Although knowledge of cervical cancer prevention was generally high and perceptions were positive among women in peri-urban Peruvian communities, our findings revealed there is a need for education on HPV infection prevalence among sexually active individuals to reduce stigma. Future research should focus on exploring experiences with follow-up and treatment associated with abnormal Pap smears, as well as perspectives from health authorities and professionals about barriers in the early detection and treatment process for cervical cancer.

## Background

Cervical cancer is a preventable and easily detectable disease, but it remains one of the leading cancers worldwide [1, 2], especially in low- and middle-income countries (LMIC) [2–5]. In South America, cervical cancer has an incidence of 23.9/100,000 and an age-adjusted annual mortality rate of 10.7/100,000 women [6, 7]. However, these averages mask heterogeneous success in controlling cervical cancer in the region, with mortality rates ranging from 6.0 (Chile) to 12.0/100,000 women (Peru), as well as heterogeneities within countries [7]. Although economic growth has been associated with a reduction in cervical cancer mortality in many countries, largely due to increased access to organized early detection and treatment (EDT) programs, [8–10] this effect has been less apparent in Latin America. A successful cervical cancer program must ensure access to screening, follow up for any abnormal results, and treatment for those who need it. EDT is a multistep process—requiring at minimum a preliminary visit for taking a sample, a return visit for results, and if the woman requires follow up, a referral for follow-up–in many cases requiring that a woman goes to make an appointment and then returns to be seen. Issues of health equity are important: Women who had lower incomes are disproportionately at higher risk of being unable to adequately do any follow-up and treatment if needed than women with higher means: they get lost in the follow up process [3, 11]. A lack of organization and quality control in EDT programs has been another factor in persistently high cervical cancer incidence and mortality [9].

Cervical cancer is the leading cause of cancer related deaths among Peruvian women, [12, 13] and in 1998, the Peruvian Ministry of Health declared that cervical cancer reduction was a national priority. The National Plan for Cervical Cancer Prevention and Control (updated in 2007) relevant at the time of this study, included a variety of provisions, including Human Papillomavirus vaccination and cervical Pap smears free of cost. Moreover, Pap smear testing is part of the first prenatal visit “package” for women in the public health system. Since this study ended, new guidelines published in 2017 by the Ministry of Health also consider HPV-testing as the preferential screening method for women between the ages of 30–49 years of age (HPV testing before the age of 30 was not recommended since many types of high-risk HPV may resolve over time). The guidelines also outlined follow up procedures depending on the results, including visual inspections using acetic acid, colposcopy, and Pap smears. Finally, women between the ages of 50–65 are recommended to have a Pap smear every three years [14].

EDT programs are available at most health facilities in Peru [15]. However, there is evidence that Peruvian women may not receive necessary and reliable information about the prevention of cervical cancer, and that knowledge of cervical cancer prevention and treatment is limited among women in lower-income Peruvian communities [19]. Beyond issues associated to knowledge, Peruvian women also report avoiding EDT for a range of reasons, including fears that Pap smear screenings are painful or cause damage (including infertility), that health professionals seem disinterested or even disrespectful, and that they fear positive test results and subsequent treatment options (or the possibility of paying for these) [3]. Misconceptions about the cost of screening and treatment, discomfort, embarrassment, fear and shame are also common [5, 16–18].

Examining the barriers to successful EDT programs requires a systems level approach to examine the multiple layers and perspectives and components affecting the quality of and complexity associated with these programs [19]. This manuscript will not focus on the multilevel complexity, but rather will focus on one important element of these programs – the women who use them: what they know, what they should know, how they feel about screening for cervical cancer, and what are their preventive practices? To expand upon and quantify findings from a previous qualitative study on this topic, the objective of this study was to quantify knowledge, social influences, attitudes, and practices of women in two low-income peri-urban neighborhoods in Lima regarding cervical cancer and its prevention [20].

## Methods

### Study design

We developed and implemented a cross-sectional survey to assess knowledge, attitudes, and practices associated with prevention of cervical cancer. We enrolled adult women living in two peri-urban neighborhoods of Lima, Peru: Pampas in the district of San Juan de Miraflores (approximate population 40,000), in 2015, and Oasis (approximate population 10,000) in the district of Villa El Salvador, in 2016. The survey included 90 questions that included sociodemographic characteristics, knowledge, attitudes, and practices associated with cervical cancer and Pap smears, including social influences which was measured by examining experiences or attitudes of those in their social circle towards cervical cancer and its prevention. The survey also included 11 open-ended questions examining general knowledge, perceptions, and beliefs, about Pap smears. Based on the study objective of quantifying knowledge, attitudes, and practices, the sample size was based on the ability to estimate the prevalence of key KAP indicators. Using prevalence estimates from a prior study in a similar low-income neighborhood of Lima over a decade earlier, [21] a sample size of 100 per community and 200 overall was estimated to be sufficient to estimate indicators to within 5- 7.5%-10.0% per community, and 5%-7.5% overall.

### Study setting

This project was conducted in two peri-urban, low-income communities, both located in the southern cone of the city of Lima: Pampas and Oasis. Both communities were primarily settled by rural–urban migrants predominantly from the highlands and from the jungle regions, Pampas over 30 years ago, and Oasis over the past 15–25 years. Around a third of women are Andean migrants, the rest were born in Lima [22–25]. Both communities began as informal settlements (squatter communities) and over time the residents undertook a long process to obtain legal titles of their land/house, which then allowed them to gain access to public services including electricity, water, and sewage systems for their homes [20, 26–28]. The infrastructure of Pampas is relatively more developed than that of Oasis; main roads are paved, and most houses have electricity and piped water [24, 29]. Oasis, settled in waves, is characteristic of other more recent settlements, having only one paved road. In Peru, generally, about 60% of the population has access to healthcare through the public Seguro Integral de Salud (SIS) (available to all Peruvians in poverty and extreme poverty, as well as to all children and pregnant women), while around 15% of the population is eligible for EsSalud (employer-based insurance), 10% through the police/military, and 8–10% via private coverage [30].

### Survey development

The survey was developed and field tested in 2015, by a group of researchers led by a Peruvian-American public health researcher with ~ 20 years’ experience working in Peru. Other members of the team include epidemiology and public health researchers who are experienced in survey development, have experience working in peri-urban Lima, and are fluent Spanish speakers. The survey was grounded in the conceptual framework of the Health Belief Model, a widely used behavior change model used in public health, and questions focused on sociodemographic characteristics; knowledge, attitudes, and practices associated with cervical cancer and Pap smears. Open-ended questions were included to obtain further depth on the main topics of interest, in the women’s words. Despite national and international bodies recommending Pap smears every three years depending on the age of a woman, the practice outcome of “Women of premenopausal age that have had a Pap smear in the last year” was selected on this tighter time frame to account for documented recall bias and low levels of accuracy in self-reported Pap-smears in low-income urban populations [31].

### Data collection process

Data collection was led by experienced community health workers who are permanently based at the research offices and live in the communities. Using non-probabilistic purposive sampling, fieldworkers recruited a maximum of three women (one woman per household) from pre-assigned geographic blocks in both Pampas and Oasis, of approximately 120 blocks in each. The geographic blocks were selected based on the relative safety of the area, and previous engagement with the research offices. Students were always accompanied by fieldworker(s) and within the selected geographic blocks the team would approach a house, introduce themselves, the project and the eligibility criteria, and ask if the person was interested in participating. Study participants were female residents who were 18 years of age or older, who were willing and able to provide verbal consent. If the person agreed to participate, verbal consent was obtained, and participants were made aware of their right to withdraw at any time. Unfortunately, the field team did not document the number of those who declined to participate. A fieldworker or student asked the questions verbatim and recorded the answer on paper copies of the survey. No incentive was offered to participate in this project and the survey took approximately 45 min to complete.

To ensure spatial distribution of the houses surveyed, the team aimed to recruit 3 houses per block. The field team used field maps to select the first house at random and then performed door-to-door sequential surveying. When houses were closed, the team moved to the house next door, until an adult female member from three households had been recruited. The field team moved to the next block if less than three households were recruited and remaining houses declined to participate or were closed. All interviews took place on the doorstep of the house or through a window, as is culturally acceptable here, and at the request of the participants.

### Data management and analysis

Interviewee responses were written in by the field team, and then quality checked by their colleagues. A database was created in Foxpro Visual 9 (I/O Technologies, Inc., Germantown, WI, USA) and data was entered twice to ensure accurate digitization. Data were exported for analysis. Open-ended questions were analyzed qualitatively by reading all the responses, developing a codebook to identify the range of themes, and then coding the responses to each question manually. Results from the open-ended questions are presented as quotes and intertwined with categorical responses in the different sections.

Two-sided t-tests were used to compare categorical knowledge, attitudes, and practice (KAP) variables while chi-2 tests were used to compare continuous variables between women by community membership (Pampas or Oasis), number of children, and level of education. Bivariate regression models were then constructed to estimate the likelihood of having had a Pap smear in the past year in relation to knowledge, attitudes, practices, and socioeconomic and demographic variables (number of children, age, and education). Correlations between knowledge, attitude, and practice (KAP) variables were weak (-0.0637, 0.3543) and scales incorporating multiple KAP variables had low internal consistency, suggesting that KAP variables reflected a range of broad constructs rather than a single latent variable. As a result, KAP variables were considered individually rather than combined into a uni- or multidimensional score. Multivariate logistic regressions models incorporating those KAP variables which had significant associations in bivariate analysis were also constructed to examine the association between various socio-demographic variables and recent Pap smear among premenopausal women (less than 41 years of age) only using a stepwise selection method. District of residence was included as a fixed effect (categorical variable). The Pearson’s Chi-2 and Hosmer–Lemeshow goodness-of-fit statistic was calculated to assess the goodness of fit of the model to the data. *P*-values of < 0.05 were considered “statistically significant” throughout. All quantitative data analysis was conducted using STATA/MP version 15 for Mac [32].

### Ethics

Institutional Review Board (IRB) approval was obtained from Tulane University School of Public Health and Tropical Medicine (#315711) and the Peruvian non-governmental organization, Asociación Benéfica PRISMA (CE0800.13). As approved by both IRB committees, all participants provided verbal consent to participate. IRB committees from both institutions approved obtaining verbal consent from participants because data were collected without unique personal identifiers and a written consent form would have been the only identifier for this minimal risk study.

## Results

A total of 224 participants were enrolled in the study. Of these, 101 were from Pampas and 123 from Oasis (Table 1). Results from both communities were combined for reporting, except where indicated. The mean age of the respondents was 34 years old. Most women reported having received at least secondary education, with approximately one third (29%) having had no or only primary education. Approximately 74% of respondents were married or lived with a partner, and homemaker was the most commonly listed occupation (63%), followed by unskilled jobs (25%). Most respondents earned more than 500 soles (~ USD 152) per month (80%) (minimum wage at the time in Peru was S/650) and a majority relied on the Seguro Integral de Salud (SIS)—which serves populations living in poverty—for health insurance (59%).

**Table 1:** Sociodemographic characteristics

Characteristics	Pampas	Oasis	Total	*P*-Value
	(n = 101)	(n = 123)	(n = 224)	
	% (n)	% (n)	% (n)	
*Education*				
None/primary only	31.7 (32)	26.8 (33)	29.0 (65)	0.426
Secondary or more	68.3 (69)	73.2 (90)	71.0 (159)	
Civil status*				
In a relationship (married/living together)	60.4 (61)	84.6 (104)	73.6 (165)	< 0.001
Single (separated, divorced, widowed, single mother)	39.6 (40)	15.4 (19)	26.4 (59)	
Number of children*				
0 children	17.8 (18)	1.6 (2)	8.9 (20)	< 0.001
1–3 children	52.5 (53)	71.5 (88)	62.95 (141)	
4 or more children	29.7 (30)	26.8 (33)	28.1 (63)	
Occupation*				
Job requiring higher education^a^	8.9 (9)	0.0 (00)	4.0 (9)	< 0.001
Job with higher education not necessary^b^	14.9 (15)	16.3 (20)	15.6 (35)	
Homemaker	52.5 (53)	71.5 (88)	62.9 (141)	
Student	12.9 (13)	0.8 (1)	6.3 (14)	
Other	10.9 (11)	11.4 (14)	11.2 (25)	
*Monthly per capita income*				
Less than or equal to 500 PEN (~ 156 USD)	18.8 (19)	20.3 (25)	19.6 (44)	0.777
More than 500 PEN	81.2 (82)	79.7 (79)	80.4 (180)	
*Type of Health insurance*^c^				
Seguro Integral de Salud (SIS)	45.5 (46)	70.7 (87)	59.4 (133)	< 0.001
EsSalud	24.8 (25)	21.1 (26)	22.8 (51)	
Private	4.0 (04)	0.0 (00)	1.8 (04)	
None	25.7 (26)	8.1 (10)	16.1 (36)	
Median age (range)*	39 (18–83)	32 (20–64)	34 (18–83)	< 0.001
Median number of people per household (range)	5 (2–13)	5 (2–17)	5 (2–17)	0.227
Median number of children (range)	2 (0–9)	3 (0–7)	2 (0–9)	0.224
Premenopausal and received a Pap smear in the last year*	9.9 (10)	45.5 (56)	29.5 (66)	< 0.001

*P < 0.05

^a^Examples include business person, administrator, health or education professional, office worker, professional work

^b^Examples include salesperson, public transportation charger, waitress, maid, gas attendant, among others

^c^Most people in Peru are covered by SIS (about 60%), another large segment of the population is covered by EsSalud which covers individuals who are salaried by any type of employer, and a small percentage are covered by private insurance. Those without insurance must represent women who made enough income to not qualify for SIS, but who did not have EsSalud

Geographical district of residence was significantly associated with civil status, household size (mean number of inhabitants per household was five in Pampas and six in Oasis), occupation, number of children, and health insurance type, but was similar for education, per capita income, number of people per household, and median income. In this bivariate analysis, we also note a statistically significant difference in the percentage of women who reported receiving a Pap smear in the last year in Pampas (9.9%) vs. Oasis (45.5%).

While most women knew when and where they should get Pap smears, open-ended responses suggested an underlying lack of knowledge about when to start getting Pap smears, and how frequently they should be repeated (Table 2). Respondents often reported that Pap smears should occur every 6 months and that having children was a prerequisite, or as one woman reported: *“…after having their children, [women] must have a Pap smear done every 6 months or every year.”* These knowledge gaps were more evident when asked about the purpose of Pap smears, with responses ranging from cervical cancer screening to screening for sexually transmitted infections or any infection, or even *“to see our ovary and see if there is anything wrong with it.”* Reasons for getting screened, in their words, focused on being told by the health facility midwife to get it done or because of pelvic pain; reasons to avoid it were because of *“fear”* or because they were *“too young”* or even *“because my mom told me I am too young to get this exam—I should get it when I have my children.”*

**Table 2:** Bivariate analysis by number of children and education levels

	Women with 0 children	Women with 1–3 children	Women with 4 + children	Low Education	High Education	Pampas	Oasis	Total
	(n = 20)	(n = 141)	(n = 63)	(n = 65)	(n = 159)	(n = 101)	(n = 123)	(n = 224)
	% (n)	% (n)	% (n)	% (n)	% (n)	% (n)	% (n)	
*Knowledge*								
Know at least one place to go to get a Pap smear	90.0 (18)	96.5 (136)	98.4 (62)	98.5 (64)	95.6 (152)	92.0 (93)*	100.0 (100)*	96.4 (216)
Cervical cancer can be prevented	100.0 (20)	95.0 (134)	92.0 (58)	87.7 (57)*	97.5 (155)*	96.0 (97)	93.5 (115)	94.6 (224)
Women should get Pap smears every 1–3 years	80.0 (16)	91.5 (129)	88.9 (56)	89.2 (58)	90.6 (144)	85.2 (86)*	94.3 (116)*	89.7 (201)
Cervical cancer can be cured	70.0 (14)	83.7 (118)	81.0 (51)	84.6 (55)	80.5 (128)	83.2 (84)	80.5 (99)	81.7 (183)
A Pap smear causes no complication	79.5 (17)	63.1 (113)	77.8 (49)	65.6 (42)**	86.7 (137)**	77.8 (77)	82.9 (102)	79.9 (179)
Women should begin getting Pap smears within 1 year after starting sexual activity	75.0 (15)	74.5 (105)	74.6 (47)	64.6 (42)*	78.6 (125)*	72.3 (73)	76.4 (94)	74.6 (167)
Pap smears are free or < 10 PEN	25.0 (5)*	65.3 (92)*	65.1 (41)*	56.9 (37)	63.5 (101)	46.0 (46)*	74.8 (92)*	61.9 (138)
Know at least one method for preventing cervical cancer	45.0 (9)	62.4 (88)	58.7 (37)	44.6 (29)*	66.0 (105)*	57.4 (58)	61.8 (76)	59.8 (134)
Know at least one correct symptom of cervical cancer	55.0 (11)	48.9 (69)	44.4 (28)	40.0 (26)	51.6 (82)	48.5 (49)	48.0 (59)	48.2 (108)
*Social Influences*								
Other women in their community are ashamed of getting a Pap smear	65.0 (13)	68.1 (96)	71.4 (45)	75.7 (49)*	66.0 (105)*	68.1 (64)	73.2 (90)	68.8 (154)
They have spoken to someone about their results and experiences	20.0 (4)**	58.9 (83)**	66.7 (42)**	55.4 (36)	58.5 (93)	69.2 (54)	64.7 (75)	57.6 (129)
Their partner approves of them getting a Pap smear	20.0 (4)**	48.2 (82)**	47.6 (30)**	40.0 (26)	56.6 (90)	56.7 (38)	70.9 (78)	51.8 (116)
Know someone that has had cervical cancer	10.0 (2)*	27.6 (39)*	46.0 (29)*	33.9 (22)	30.2 (48)	30.7 (31)	31.7 (39)	31.3 (70)
Other women in their community talk about cervical cancer	15.0 (3)**	23.4 (33)**	49.2 (31)**	33.9 (22)	28.3 (45)	29.3 (29)	30.9 (38)	29.9 (67)
*Attitudes*								
Women feel comfortable in the appointment with their OBGYN	55.0 (11)*	86.5 (122)*	85.7 (54)*	78.5 (51)	85.5 (136)	79.8 (79)	88.5 (108)	83.5 (187)
Women view Pap smears as being of very high importance (5 on a scale of 1 to 5)	75.0 (15)	72.3 (102)	63.5 (40)	55.4 (36)*	76.1 (121)*	63.0 (63)*	76.4 (94)*	70.1 (157)
Women that fear knowing the result of their Pap smear	45.0 (9)	42.6 (60)	41.3 (26)	50.8 (33)	39.0 (62)	40.4 (40)	44.7 (55)	42.2 (95)
*Practices*								
Women of premenopausal age that have had a Pap smear in the last year	10.0 (2)**	38.3 (54)**	15.9 (10)**	13.9 (9)**	35.9 (57)**	9.9 (10)**	45.5 (56)**	29.5 (66)

*P < 0.05

**P < 0.001 for differences between women with 0, 1–3, and 4 + children **P < 0.001 for differences between women with less than secondary school versus secondary school or high

Most women demonstrated accurate knowledge of cost, complications, and the preventability and curability of cervical cancer. However, open-ended responses about the preventability and curability of cervical cancer were mixed, with only some acknowledging that cervical cancer could be cured if caught in time. Other responses focused on promiscuity, male sexual habits or infidelity, early sexual initiation, rape, and a lack of hygiene as the primary way of contracting cervical cancer. When asked if there was something you could do to influence the possibility of having cervical cancer, one respondent noted that: *“The husband transmits it, he is not hygienic, it’s the promiscuity of the man.”* Another mentioned that cervical cancer is hereditary.

A majority of women viewed Pap smears as being of very high importance (70%), felt comfortable with their healthcare provider (84%), and said that they themselves had spoken to someone about their Pap smear results and experiences (58%). However, 69% also reported that other women in their community were ashamed to get Pap smears, reported knowing someone who has had cervical cancer (31%) or reported that women in their community talked about cervical cancer (30%). Almost half of women feared knowing the results of their Pap smears (42%). Finally, 30% of premenopausal age women had received a Pap smear in the last year, which would imply that up to 90% of women may get a Pap smear once every three years—but we did not ask about Pap smears over this longer time scale. When asked in open ended questions why they hadn’t received a Pap smear within the last 12 months, multiple women said they were afraid, while others specified undesirable outcomes including urinary tract infections, complications while pregnant, and bleeding or discomfort after their last Pap smear.

Significant associations were observed between both the level of education, number of children, district of residence and various knowledge, social influence, attitude and practice variables (Table 2). Knowing that women should begin getting Pap smears within 1 year of starting sexual activity, knowing that Pap smears cause no complications, knowing other women in their community are ashamed of getting Pap smears, and holding Pap smears in high importance were some of the variables statistically significantly associated with level of education. Knowing the correct price of a Pap smear, knowing someone who had cervical cancer, feeling comfortable with your healthcare provider, and speaking to someone about their Pap smear results and experience, among other variables, were statistically significantly associated with number of children. Knowing at least one place to get a Pap smear, knowing that women should get a Pap smear every 1–3 years, and holding Pap smears in high importance were some of the statistically significant associations with district of residence. Among the subset of women of premenopausal age (41 years of age or younger), having a Pap smear within the last year was associated with education, number of children, and district of residence.

Multivariate analysis (Table 3) considered the effects of respondent level of education, civil status, age, district of residence, and number of children. Women with post-secondary education were five times more likely to have received a Pap smear in the last year than those with primary education or less, women in relationships were nearly six times as likely to get Pap smears than those who were single, and women in Oasis were more likely to have received a Pap smear in the past year as those living in Pampas. None of the knowledge, social influences, or attitude variables were associated with premenopausal women having received a Pap smear in the past year. Monthly per-capita income was explored as a covariate but made no significant changes in the adjusted model. Pearson’s Chi-2 and Hosmer–Lemeshow Chi-2 test for goodness of fit both resulted in p-values of 0.2193 and 0.4370 respectively, suggesting the model fits the data relatively well.

**Table 3:** Multivariate logistic regression

	Received Pap in past year (N = 151)			
	Adjusted OR (95% CI)	Unadjusted OR (95% CI)	Received Pap in past year %(n)	Did Not Receive Pap in past year %(n)
*Education*				
No education/Primary education only	Ref	Ref	34.62 (9)	65.38 (17)
Secondary Education	2.23 (0.81, 6.17)	1.70 (0.68, 4.29)	47.44 (37)	52.56 (41)
Technical School/University	5.00 (1.36, 18.46)	1.40 (0.52, 3.78)	42.55 (20)	57.45 (27)
*Civil status*				
Single (Single, Divorced, or Widowed)	Ref	Ref	17.65 (6)	82.35 (28)
In a relationship (Married, Cohabitating)	5.93 (1.73, 20.26)	4.91 (1.89, 12.74)	51.28 (60)	48.72 (57)
*Age*				
Under 25	Ref	Ref	41.67 (20)	58.33 (28)
26–35	0.63 (0.23, 1.71)	0.99 (0.47, 2.09)	41.42 (29)	52.57 (41)
36–41	1.55 (0.44, 5.42)	1.49 (0.61, 3.62)	51.52 (17)	48.48 (16)
*District/City*				
Oasis	Ref	Ref	57.14 (56)	42.86 (42)
Pampas	0.18 (0.07, 0.45)	0.17 (0.08, 0.39)	18.87 (10)	81.13 (43)
*Number of children*				
No Children	Ref	Ref	10.53 (2)	89.47 (17)
1–3 Children	1.69 (0.24, 11.75)	8.50 (1.87, 38.59)	50.00 (54)	50.00 (54)
4 or more Children	0.94 (0.10, 8.95)	6.07 (1.14, 32.41)	41.67 (10)	58.33 (14)

## Discussion

This study assessed knowledge, attitudes, practices, as well as social influences associated with having received a Pap smear in the past year to inform topics that require dissemination in health facilities. Women were generally familiar with Pap smears and cervical cancer, understood the importance of Pap smears, and displayed high knowledge of the logistics associated with cervical cancer screening in quantitative responses. Moreover, 30% of women reported being screened in the past year; considering current recommendations of Pap smears every three years, this implies that up to 90% of women in these communities are receiving their screening as needed. However, a number of factors suggest that the true prevalence of screening is likely to be somewhat lower than 90%, including the potential for overreporting associated with telling the research team what they thought was the correct response (social desirability bias), as well as the possibility that some women were receiving Pap smears more often than every three years due to relatively recent changes in the recommendation (Peru shifted from recommending screenings every year to every three years in 2008) or for other reasons. The fact that we noted multiple differences in socio-economic status, as well as reported knowledge and attitudes, between women with and without a recently reported screening, also suggests that screening in this community is not ubiquitous and that appropriately targeted interventions could increase the screening prevalence. The open-ended questions included in our survey also highlighted knowledge gaps that point to some underlying confusion and stigma towards Human Papiloma Virus (HPV) infection and screening amongst women in these communities. Not only did they frequently link the need for Pap smears to childbearing, they also associated HPV infection with poor hygiene, early sexual initiation, promiscuity, and infidelity, particularly on the part of male partners. Stigma associated with HPV infection has been described in the literature; community health education focusing on how common HPV infection is among sexually active adults might mitigate the stigma associated with this [33].

Although our survey focused on factors associated with screening, we also acknowledge that this is only a first step in the EDT processes, and must be followed by appropriate return of results, and, if necessary, referral-aspects of the EDT process that are likely to be more complex and time consuming for women to follow. Therefore, any activities to increase the number of women who receive a Pap smear once every three years should also be accompanied by efforts to ensure that the timely follow up and treatment that follow the screening are adequate.

Our results align with existing literature throughout Peru and the world. A qualitative study using focus groups with women from three geographic regions of Peru found that about two thirds of women knew that they should receive Pap smears every year, and about half knew to start receiving these Pap smears shortly after commencing sexual activity [18]. Our study in the capital city of Lima showed that 90% of women knew that Pap smears should be done every 1–3 years, and 75% of women correctly identified that they should be receiving Pap smears within a year after starting to engage in sexual activity.

Bivariate analysis also suggests prior reports that higher levels of educational attainment has been associated with increased knowledge of cervical cancer and Pap smears [34–36] and that number of children is highly predictive of receiving a Pap smears, likely attributable to the policy of cervical cancer screening as part of antenatal care [5, 17, 18]. As expected, knowledge variables were more likely to be associated with level of education, but interestingly, variables associated to measures of social influences related to Pap smear seeking behaviors were more commonly associated with having children (vs. not) or a higher number of children. Though further research is required to examine why measures of social influence were associated with number of children, we have a few thoughts on this. Although the latest Ministry of Health and EsSalud guides we could access (2011) established that Pap smears should be done every three years or annually starting at the age of 21 and 18 respectively, conversations with women and evidence in the literature reveals that some women are told or believe one should start getting their Pap smears once they have had children [18]. Hence, it is possible that women start thinking about Pap smears and/or cervical cancer once they start being tested, which for most occurs during antenatal care. Finally, it is also possible that once women have children, they start talking with other mothers about various items related to general health—their children’s health, nutrition, and possibly women’s health—whereas prior to having children, the focus of many women’s conversation might be different. This would be in line with open-ended responses from multiple women that suggest a strong belief that Pap smears are something women should do once they have had, or are done having, children and suggest that although women knew they should get Pap smears on becoming sexually active, they were likely to delay actually starting until they had had a child.

In unadjusted models, civil status, district/city of residence, and having 1–3 children were significantly associated with premenopausal women receiving a Pap smear in the past year, whereas in adjusted multivariate models, education, civil status, and district were highly associated with premenopausal women receiving a Pap smear in the past year, a finding that is consistent with accepted literature [17, 37–40]. However, despite the level of education and number of children being significantly associated, the number of children was not associated with receiving a Pap smear within the last year, suggesting that other factors beyond education are influencing Pap smear behavior.

Geographical effects (location of residence) were observed for premenopausal women receiving a Pap smear in the last year in both bivariate and multivariate analysis. This suggests that access to Pap smear was unequal between the communities. In fact, Oasis in the district of Villa el Salvador was established much more recently than Pampas in the district of San Juan de Miraflores, is a poorer shantytown, and has less infrastructure, [41] yet the women in this community are significantly more likely to report having received a Pap smear in the past year. However, women in Oasis tended to be more educated, more likely to be in a relationship, and have children, than women in Pampas, which may explain some of the differences. Moreover, data for this study was collected over two summers with sampling in Pampas being conducted in the summer of 2015 while Oasis was sampled in the summer of 2016. The time-lapse in data collection may have allowed for certain events or external factors to affect one community and not the other (i.e., someone in one community died of cervical cancer, or there was an educational event in one community vs. the other, or there is a friendly health provider in one health post in one community vs. the other), ultimately skewing results and possibly explaining some of the discrepancies between our findings and accepted literature.

This descriptive, cross-sectional, multi-method study was a snapshot of women’s knowledge, attitudes, and practices around cervical cancer screening and their social influences in two low-income neighborhoods in the peri-urban areas surrounding Lima, and is based on a small convenience sample. One limitation is that the survey instrumentation to assess knowledge, attitudes, social influences, and practices was not designed to develop uni- or multidimensional scales to assess these constructs. Efforts to develop some validated scale to assess these constructs in the future may prove valuable. Also, we understand an EDT program has many components—screening, follow up, and treatment—and that this study design only allowed us to examine screening. Locally-tailored instruments to characterize knowledge, attitudes and practices should include items to examine follow up and treatment experiences of women, and to characterize community trust and confidence in the healthcare providers and systems generally. Finally, in order to obtain a better understanding of this complex topic and aware that women’s knowledge, attitudes and practices are only one component of the screening process, we strongly believe that future research must examine the EDT system and its bottlenecks from different perspectives—health providers, labs, health authorities.

## Conclusion

Early detection and treatment programs for cervical cancer are complex: there are many factors at many levels that affect the success of the screening programs, from access, to types of screening, to barriers to diagnostic and follow up, among many others, and all of this influence the effectiveness of the program. This study highlights one element of many others: variations in levels of knowledge, attitudes, and practices among women in peri-urban communities surrounding Lima, Peru, that impact the likelihood of reporting a recent Pap smear among these populations. We also find that knowledge and attitudes towards cervical cancer, its prevention, and cervical cancer screening are highest among women who have had children—reflecting the fact that many women first learn about the importance of early screening when they start receiving antenatal care, where it is offered as part of the program. This suggests a need for messaging to promote cervical cancer screening among women who delay childbearing or do not have children. Disagreement between knowledge in open-ended responses, and quantitative measures of knowledge and practice (having received a Pap smear), also suggests deeper confusion within these populations that was not captured by standard knowledge, attitude, and practice questions. Moreover, to reduce stigma associated with HPV infection, community health education and counseling could be helpful. Future studies must focus on other elements of early detection and treatment for cervical cancer to help us understand the complex system and find leverage to develop the most appropriate and locally-designed interventions to reduce cervical cancer morbidity and mortality in Peru.

## Data Availability

The datasets used and/or analyzed during the current study are available from the corresponding author on reasonable request.

## Abbreviations

KAP: Knowledge, attitudes, and practices
GDP: Gross domestic product
SIS: Seguro Integral de Salud
